# Molecular and Physiological Characterization of Fluoroquinolone-Highly Resistant *Salmonella* Enteritidis Strains

**DOI:** 10.3389/fmicb.2019.00729

**Published:** 2019-04-09

**Authors:** Sinisa Vidovic, Ran An, Aaron Rendahl

**Affiliations:** Department of Veterinary and Biomedical Sciences, University of Minnesota, Saint Paul, MN, United States

**Keywords:** *Salmonella*, quinolone resistance, multidrug resistance, AcrAB efflux pump and small-colony variant phenotype, OmpF porin

## Abstract

Four clinical isolates of *Salmonella* Enteritidis, susceptible to ciprofloxacin, and their spontaneous ciprofloxacin resistant (MICs from 8 to 16 μg/mL) and highly resistant (MIC 2048 μg/mL) mutants were used to gain an insight into the dynamics of development of fluoroquinolone (FQs) resistance in *S.* Enteritidis serovar. The first two high-frequency (i.e., mutations that occurred in each tested strain) mutations occurred in the *gyrA*, resulting in amino acid substitutions S83Y and S83F as well as D87G. Amino acid substitution D87G was significantly associated with the highly resistant mutants. Another high-frequency mutation, deletion in the *ramRA* intergenic region, was determined among the same group of highly resistant mutants. More importantly, each of these deletion mutations affected the RamR binding site. The effect of one 41 bp deletion mutation was empirically tested. The results showed that the deletion was responsible for resistance to ceftiofur and amoxicillin/clavulanic acid and decreased susceptibility to azithromycin and tetracycline. Performing gene expression assays across all ciprofloxacin susceptible groups, we found a consistent and significant upregulation of the *ramA*, *acrB*, and *tolC* (efflux pump associated genes) and downregulation of *ompF* (porin), clearly illustrating the importance of not only efflux but also porin-mediated permeability in the development of FQs resistance. Our data also showed that *S*. Enteritidis could acquire multiple mutations in QRDR region, further resulting in no up regulation of the *ramA, acrB and tolC* genes. These QRDR mutations and no activation of the AcrAB efflux pump seem to preserve the fitness of this organism compared to the *S*. Enteritidis strains that did not acquire multiple QRDR mutations. This report describes the dynamics of FQ-associated mutations in the highly resistant in FQ mutants in *S*. Enteritidis. In addition, we characterized a deletion in the *ramRA* integenic region, demonstrating that this frequent mutation in the highly resistant FQ mutants provide resistance or reduce susceptibility to multiple families of antibiotics.

## Introduction

Non-typhoidal *Salmonella* (NTS) is a major zoonotic pathogen worldwide (Bangtrakulnonth et al., 2004; Scallan et al., 2011). Infections caused by this pathogen have been mainly associated with gastroenteritis, an acute self-limiting intestinal infection. However, it has been shown that in different regions of the world, especially in places with high percentages of immunocompromised populations, NTS is a frequent cause of bacteremia (Graham, 2010; Reddy et al., 2010; Okoro et al., 2012), an invasive life-threatening extra intestinal infection. Once acquired, this invasive salmonellosis may result in a fatality rate of 20% (Gordon, 2008). Multidrug resistance and in particular resistance to fluoroquinolones (FQs), a potent broad-spectrum family of antibiotics used for the primary treatment of invasive salmonellosis, play a key role in the treatment failure (Pers et al., 1996; Rupali et al., 2004).

Fluoroquinolones resistance in NTS can be acquired through transmissible quinolone-resistance mechanisms (Martinez-Martinez et al., 1998; Redgrave et al., 2014; Hooper and Jacoby, 2015). Transmissible quinolone-resistance occurs via the horizontal transfer of plasmids, which carry a family of *qnr* genes (i.e., *qnrA*, *qnrB*, *qnrS*, *qnrC*, and *qnrD*)—also known as plasmid-mediated quinolone resistance (PMQR) genes. It has been shown that the PMQR genes confer modest resistance against FQs (Martinez-Martinez et al., 1998). *Salmonella* spp., most commonly vertically acquired resistance to FQs through *de novo* mutations, which occurs in the quinolone resistance-determining region (QRDR) of the *gyrA* and *parC* genes (Piddock et al., 1998; O’Regan et al., 2009; Ricci and Piddock, 2009) and in genes encoding the *acrAB*-*tolC* efflux system (Baucheron et al., 2004) as well as in genes that encode regulators of the efflux system (Abouzeed et al., 2008; O’Regan et al., 2009; Blair et al., 2015). Vertically acquired mutations play a critical role in antimicrobial treatments, as these mutations occur quickly under the selective pressure of the drug, resulting in a high level of resistance against FQs, which may subsequently lead to antimicrobial treatment failure. The vertical evolution of resistance-conferring mutations is very complex. The outcome, antimicrobial susceptibility, depends on a sum of the interactions between antagonistic and synergistic resistance-conferring mutations rather than on an independent effect of a single mutation (Hartl, 2014). Understanding the development of *de novo* mutations, their interactions, and physiological adaptation of the bacterial organism to the selective pressure imposed by the drug is critically important in antimicrobial stewardship programs.

In this study, we examined the development of the resistance and the high-level resistance to FQs using four ciprofloxacin susceptible *Salmonella enterica* serovar Enteritidis (*S*. Enteritidis) isolates and their spontaneous ciprofloxacin resistant (MIC ranging from 8 to 16 μg/mL) and highly resistant (MIC 2048 μg/mL) mutants. The aim of this study was to determine the type and dynamics of the mutations and the overall physiological adaptations associated with the development of extremely high resistance to ciprofloxacin.

## Materials and Methods

### Strains of *S*. Enteritidis

Out of 88 *S*. Enteritidis strains, implicated in human and avian infections, we selected four strains that exhibited the most profound susceptibility to ciprofloxacin. The following four *S.* Enteritidis strains: A-5 from Texas (MIC 0.015 μg/mL), A-7 from Pennsylvania (MIC 0.06 μg/mL), A-21 from Iowa (MIC 0.03 μg/mL), and A-33 from Wisconsin (MIC < 0.0009375 μg/mL) were selected for the study. All four strains were implicated in the primary infections of avian hosts. The strains were received from the National Veterinary Services Laboratories (NVSL), Ames, IA. The *S*. Enteritidis strains were diagnosed at the NVSL using standard microbiological and serological methods for *Salmonella* identification and classification. The strains were checked for their purity upon their arrival by plating them on Luria-Bertani (LB) agar (Difco) plates, followed by an overnight incubation at 37°C. After confirmation of the strains’ purity, they were stored at −80°C in LB broth (Difco) with 10% glycerol.

### Antimicrobial Susceptibility Tests

Four *S.* Enteritidis strains, A-5, A-7, A-21, and A-33, were examined for their antimicrobial susceptibilities using Sensititre CMV3AGNF plates (TREK Diagnostic Systems, Cleveland, OH, United States). The Sensititre plates each contained 14 antimicrobial agents dosed in 96 wells at appropriate dilutions, as specified by NARMS (National Antimicrobial Resistance Monitoring System) of the CDC. Each well of the Sensititre microtiter plate was inoculated according to the instructions of the manufacturer, followed by incubation at 37°C for 18–22 h. The minimal inhibitory concentration (MIC) breakpoints were determined according to the National Committee for Clinical Laboratory Standards (NCCLS) M07-A10 (Clinical and Laboratory Standards Institute [CLSI], 2015a) and M100-S25 (Clinical and Laboratory Standards Institute [CLSI], 2015b).

### Induction of FQ Resistance and Selection for Resistant and Highly Resistant Mutants

To obtain this collection, we exposed each parental strain at its mid-exponential growth phase to 0.02 μg/mL of ciprofloxacin, followed by an overnight incubation period. After the overnight incubation, ciprofloxacin-challenged cultures were inoculated (1:100) into LB broth, containing a twofold higher concentration of ciprofloxacin compared to the previous ciprofloxacin challenge. Further, a series of twofold increase ciprofloxacin concentrations (from 0.04 to 2.4 μg/mL) challenges were undertaken, and a selection of the ciprofloxacin resistant mutants took place on LB agar plates with 4 μg/mL of drug concentration. Similarly, after a series of twofold increase ciprofloxacin concentrations (from 8 to 32 μg/mL) the highly resistant strains were selected on LB agar plates, which contained 40 μg/mL of ciprofloxacin.

### Minimal Inhibitory Concentration (MIC) Determinations

The MICs of ciprofloxacin for the parental strains and their resistant and highly resistant spontaneous mutants were determined using broth macrodilution method, as described by the NCCLS M07-A10 (Clinical and Laboratory Standards Institute [CLSI], 2015a) and M100-S25 (Clinical and Laboratory Standards Institute [CLSI], 2015b).

### Cross-Resistance Assay

After selecting the resistant, A-5 (R), A-7 (R), A-21 (R), and A-33 (R) and the highly resistant mutants, A-5 (HR), A-7 (HR), A-21 (HR), and A-33 (HR), they were examined for any additional acquired antimicrobial resistance phenotype, which is not relevant to FQ class of antibiotics. The susceptibility of the resistant and the highly resistant strains was determined using Sensititre CMV3AGNF plates, as described above.

### *gyrAB*, *parCE*, *acrAB-tolC*, *ramAR*, *rpoE*, *cpxR, ompFC*, and *lpxA* Full-Length Genes Sequencing and Single Nucleotide Polymorphisms (SNPs) Analysis

The list of the primers used for the full-length gene sequencing of *gyrA*, *gyrB*, *parC*, *parE*, *acrA*, *acrB*, *tolC*, *ramA*, *ramR*, *rpoE*, *cpxR*, *ompF*, *ompC*, and *lpxA* is shown in Table 1. The primers for *acrA*, *acrB*, *tolC*, *ramA*, *ramR*, *rpoE*, and *cpxR* are designed to target the promoter regions of these genes as well. The amplicons were generated by Platinum *Taq* DNA polymerase (Thermo Fisher Scientific) and were prepared for DNA sequencing by the Prism BigDye Terminator cycle sequencing kit (Applied Biosystems, Foster City, CA, United States). The nucleotide sequences on both strands were determined using an ABI 3730 x 1 DNA analyzer (Genomics Center, University of Minnesota, Minneapolis, MN, United States). Each strand was checked and then aligned with its complementary strand. A consensus DNA sequence was obtained using Clustal Omega (Larkin et al., 2007). The annotated DNA sequences were exported into Molecular Evolutionary Genetics Analysis (MEGA) version 7 (Tamura et al., 2007) for the identification of non-synonymous single nucleotide polymorphisms (nsSNPs) within the coding regions and SNPs within the promoter regions of *acrA*, *acrB*, *tolC*, *ramA*, *ramR*, *rpoE*, and *cpxR*. Nucleotide sequence translation was carried out using EMBOSS Transeq (Kearse et al., 2012) (the European Molecular Biology Laboratory–European Bioinformatics Institute; Hinxton, Cambridge, United Kingdom).

**Table 1:** Primers used for the amplification and full gene sequencing.

Gene	Purpose	Name of primer	Forward primer sequence (5′ –3′)	Name of primer	Reverse primer sequence (5′–3′)	Amplicon size (bp)
*gyrA*	PCR	*gyrA* F	CGCCAATAAACGCCAAGACC	*gyrA* R	AGCGGTAAATGACGTAGCCC	2769
	Sequencing	F-1	GGTGAACCTCAACGACGGC	R-1	ACTGCGATACCGGAAGAACC	
	Sequencing	F-2	CTATGACGGTACGGAAAAAAT	R-2	CGGTGGCGCACGAACGCTGAAATG	
	Sequencing	F-3	GACCCAGCTACAGGTTTCCTTC	R-3	CTGGCTAATCAGATCTTCGATATT	
	Sequencing	F-4	ATCCGCGAAGAGATGGAGTTA	R-4	GACGGCGTCTTCTTTGAAGC	
*gyrB*	PCR	*gyrB* F	TAGCGCTGAACACGTTATAGAC	*gyrB* R	GCCACTCAGCGCTTTAGAGAT	2584
	Sequencing	F-1	CAGCGAGATGGCAAAATTCAC	R-1	CGCAATCAGACCTTCACGGG	
	Sequencing	F-2	TGGACAAAGAAGGCTACAGCA	R-2	CTTGTCCGGGTTGTACTCGT	
	Sequencing	F-3	TCGACAAGATGCTTTCCTCCC	R-3	CATCGAACTTCCACTGACTGC	
	Sequencing	F-4	GCTGGGTGAATGCGCTG	R-4	GCCAGAAACGTACCATCGTG	
*parC*	PCR	*parC* F	CCACCTGTCTCACGGTTTGT	*parC* R	GGCCGGATAAGACGCTACTG	2419
	Sequencing	F-1	AATACGCCGAGCTGCTGTTA	R-1	GTGGTGGCGAACAGATGGTT	
	Sequencing	F-2	ACCCGACGCGTTTAGTGATT	R-2	GCTTTAGCTTCTTCGCGCTC	
	Sequencing	F-3	GTCCGAACGCAAAATGAATACC	R-3	TCTTCAATCACCAGCGGCG	
	Sequencing	F-4	GCTACGGCTTCGTTTGTACG	R-4	CGCCGGTAACATTTTCGGTT	
*parE*	PCR	*parE* F	GCATTCACGGGCTTCGAAGATT	*parE* R	ATGCGCAAGTGTCGCCATCA	2060
	Sequencing	F-1	CGCGACGGTCAGGTCTATAA	R-1	CGACAGACGCTCTTTGGTCT	
	Sequencing	F-2	ATACCGCAATATTCTGCCGC	R-2	GACTCAGATCGTCGCTGTCC	
	Sequencing	F-3	CTTCCGATGAAGTGCTGGC	R-3	AATGGACGCTGGTTCCAGTA	
*acrA*	PCR	*acrA* F	AAACGCAGGGCCACATCCAG	*acrA* R	TGATGGCGATCACCCACGCA	1230
	Sequencing	F-1	GATTGATCCTGCGACCTACCA	R-1	GGCGCGCAAAGTAATAGACC	
	Sequencing	F-2	AAACAGGAAAACGGCAAAGCG	R-2	TTCACCGTCAGTTCAGCGAT	
*acrB*	PCR	*acrB* F	GCGCACAGGTTAAAGTACA	*acrB* R	AATAATCAATCTAACAATAAGCGT	3295
	Sequencing	F-1	CGTGAGCGTTGAGAAGTCC	R-1	GGCGTGGTGTCATACGGG	
	Sequencing	F-2	TTGGCATCAAACTGGCTACC	R-2	CGTCAGGATCAGCGCGAC	
	Sequencing	F-3	CAACCGGGGCAATTTATCGT	R-3	ATTTGTGAAAACGCTGCGG	
	Sequencing	F-4	CCGGTATTGCATTCGTGTCG	R-4	TTATAGCGTTCCAGACGCGG	
	Sequencing	F-5	AACGACTGGTACGTTCGTGG	R-5	ACCCAATGGTTGTGAGCAGG	
	Sequencing	F-6	CTATCCCGTTCTCCGTAATG	R-6	AGAGGTACGGCTGATCGGA	
*tolC*	PCR	*tolC* F	CTGCTGTTTTATCACCCTTGTGG	*tolC* R	TCAGGCGCGGCTTTATGAC	1692
	Sequencing	F-1	CTTTCCTATACCCAGGCGCA	R-1	CTTTCCAGCTGTTCGCTTGC	
	Sequencing	F-2	TGGGATGGTTAACTCGCAGG	R-2	TTCGCGGTATTGAGGATCAGC	
*ompC*	PCR	*ompC* F	CCGTTGATTTTAAAAGTTTCGT	*ompC* R	GGACGCAAGCGTATATCAAA	1301
	Sequencing	F-1	GATGGCGACCAGACCTACAT	R-1	TACTGCGCTGCCAGATAGAT	
	Sequencing	F-2	GGCGCTATCACCACGTCTAAA	R-2	TTCAGTCTGGTTGCCCTGAA	
*ompF*	PCR	*ompF* F	ACCAAATCTTTATCTTTGTAGCA	*ompF* R	CCTGTTTTTGAAAGACGCAC	1245
	Sequencing	F-1	CTAAAGCAGACCGCGCTGA	R-1	AACGGCCCACGATTCTGC	
	Sequencing	F-2	AGCGTACAGCAACAGCAAGC	R-2	CGACCATAATCGATTGAACCC	
*ramRA*	PCR	*ramRA* F	CATCGTACTGTGGGCCGAA	*ramRA* R	TGGTTTCTGTTGCTCGGCG	
	Sequencing	F-1	ACGAGTCATCATTTTGGCATC	R-1	AAGCATAGTAATAACCACACAAA	
	Sequencing	F-2	TTGACGGCGTATCTTTGCTTT	R-2	GCGACCAAAGATGAGCTGATTA	
*rpoE*	PCR	*rpoE* F	AACATGGTTGCGGCAGATTAG	*rpoE* R	ACGTTTCGCCATCCATCAAAG	1014
	Sequencing	F-1	GAGCGAGCAGTTAACGGACCA	R-1	GGAAACCAGACTCGCCACT	
*cpxR*	PCR	*cpxR* F	TAACTTTGCGCATCGCTTGC	*cpxR* R	ATCGAGCTTGGGCAACATCA	917
	Sequencing	F-1	CGCGGACGACTATTTACCCA	R-1	GGCGCAAAATAGCCCTGATG	
*lpxA*	PCR	*lpxA* F	CGTGGCCTGACCCGCTTTA	*lpxA* R	CACGAATTAAGCCTGCGCCA	1128
	Sequencing	F-1	GTCGCACATGATTGTACGGT	R-1	TCATCGACTGATACGTGCCCC	

### Gene Expression Assay

Overnight cultures of the susceptible, resistant, and highly resistant *S*. Enteritidis A-5, A-7, A-21, and A-33 strains were diluted 1/100 in 100 mL of LB and grown at 37°C with constant shaking at 190 rpm to optical density at 600 nm of 0.5. These mid-exponential growth phase cultures were exposed to ciprofloxacin at a final concentration of 0.010 μg/mL. The cultures were additionally incubated for 30 min and harvested by centrifugation. The total RNAs were extracted using the RNeasy Mini kit (Qiagen), following the manufacturer’s instructions. The synthesis of complementary DNA (cDNA) was carried out using iScriptTM Reverse Transcription (Bio-Rad Laboratories, Inc. Hercules, CA, United States). Quantitative PCR was performed using Power SYBR green master mix kit (Applied Biosystems). The primers used to detect transcripts of *ramA*, *ramR*, *acrA*, *tolC*, *rpoE*, *cpxR*, *ompA*, *ompW*, *ompF*, *ompC*, and *slyB* are listed in Supplementary Table S1. The *gapA* gene was selected as an internal reference control and the data were reported as the fold change relative to the levels in the susceptible strains using the comparative *C_T_* method (Schmittgen and Livak, 2008).

### Complementation Assay

The *ramRA* sequence, including its intergentic region, was amplified by PCR using genomic DNA of *S*. Enteritidis 5-A highly resistant strain, Q5 High-Fidelity DNA polymerase (New England BioLabs, Ipswich, MA, United States), and the primers *ramRA* F (5′-GCG GGA TCC GAC AGT GAT GTT CAG TGA AC-3′) and *ramRA* R (5′-TCA GTC GAC CTC TTG CTC GGC GCG CTG GA-3′); the *Bam*Hl and *SaI*l sites are underlined. The *ramRA* fragment was double digested and cloned between the *Bam*Hl and *Sal*I restriction sites of the low copy expression vector pTrc99A (Life Science Market). The recombinant pTrc99A vector was sequenced and an insertion of the *ramRA* haplotype of S. Enteritidis 5-A highly resistant strain was confirmed. The *S*. Enteritidis 5-A susceptible strain was transformed with the recombinant pTrc99A vector and also with an empty pTrc99A vector, respectively. Both *S*. Enteritidis A-5 susceptible strains, complemented with the recombinant and non-recombinant pTrc99A vectors, were tested for susceptibility to the panel of 10 antimicrobial agents, as described above.

### Growth Curve Assay

The difference in the growth kinetics between the parental ciprofloxacin susceptible strain and its spontaneous highly resistant mutant with a small colony variants (SCVs) phenotype was determined by measuring biomass at 600 nm. The overnight cultures of the parental and highly resistant mutant strains were diluted and normalized to an optical density equivalent to 0.5 McFarland standard (1.5 × 10^8^ CFU/mL). These normalized cultures were used to inoculate 50 mL of freshly prepared LB, followed by incubation at 37°C with shaking at 180 rpm. The OD_600_ values were measured every 60 min for 6 h. In addition, the differences in the growth kinetics between the parental strains A-5, A-7, A-21, and A-33 and their resistant mutants were measured. This growth assay was carried out with addition of 0.005 μg/mL of ciprofloxacin as described above.

### Statistical Analysis

To test for differences in gene expression between resistant and highly resistant strains, compared with their parental susceptible strains, *t*-tests were performed for each strain/gene/resistance combination. To better estimate variability, variance was pooled for data from the same strain. Additionally, *p*-values were adjusted for multiple comparisons using the Bonferroni–Holm correction, again, within each strain separately. All tests were performed on the ddCt scale, and results transformed to fold change for presentation. Growth curves kinetics were analyzed by CoStat version 6.4 software (Co-Hort Software, Monterey, CA, United States) using homogeneity of linear regression slopes method to test for significant (*p* < 0.05) differences.

**Table 2:** Summary of MICs, amino acid substitutions, deletion mutations in the *ramRA* intergenic region and gene expression fold changes for the *ramA*, *acrB* and *ompF*.

	MIC(ug/mL)	Amino acid substitutions		Deletion mutations	Gene expression fold change		
				
	Ciprofloxacin	GyrA	ParC	RamRA	*ramA*	*acrB*	*ompF*
A5-S	0.015				1	1	1
A5-R	16	S83Y; D87G	S80R		1.3	2.7	−2.9
A-5-HR	2048	S83Y; D87G	S80I	41 nt + 1 mutation	80	6.6	−7.7
A-7-S	0.06				1	1	1
A-7-R	16	S83Y			11	3.6	−25
A-7-HR	2048	S83Y; D87G	G78D	9 nt	83.3	10.2	−3.8
A-21-S	0.03				1	1	1
A-21-R	8	S83Y		11 nt	72.2	8.3	−6.7
A-21-HR	2048	S83F; D87G		12 nt +1 mutation	205.8	14.5	−16.7
A-33-S	<0.0009				1	1	1
A-33-R	16	S83F			35.8	5.6	−100
A-33-HR	2048	S83F; D87G		4 nt	22.6	7.4	−43.3

### Nucleotide Sequence Accession Numbers

Nucleotide sequences of each gene haplotype were deposited in GenBank under accession numbers MH933946 to MH933963. Also, accession numbers for six *ramAR* haplotypes, including their intergenic regions, range from MK024405 to MK024410.

## Results

### Selection and Characterization of Spontaneous Ciprofloxacin-Resistant and Highly Resistant *S*. Enteritidis Mutants

Four clinical strains of *S*. Enteritidis with a high level of ciprofloxacin susceptibility, A-5 (MIC 0.015 μg/mL), A-7 (MIC 0.06 μg/mL), A-21 (MIC 0.03 μg/mL), and A-33 (MIC < 0.0009 μg/mL), were used to create a collection of spontaneous ciprofloxacin resistant and highly resistant mutants. Following the selection of the ciprofloxacin resistant and highly resistant mutants, we tested these two groups of mutants for their MIC values to ciprofloxacin. The resistant mutant strains exhibited MIC values ranging from 8 to 16 μg/mL, while their highly resistant counterparts achieved an MIC of 2048 μg/mL, indicating much higher MIC values compared to those of the resistant mutant strains (Table 2).

### Exposure of *S*. Enteritidis to Ciprofloxacin Leads to the Development of Cross-Resistance

To determine the potential side effect of the ciprofloxacin treatment on antimicrobial susceptibility of the spontaneous mutants to antibiotics that are not related to the family of FQs, we carried out the antimicrobial susceptibility tests for the parental strains and their spontaneous resistant and highly resistant mutants using ten, non-FQs-related antibiotics. The susceptibility tests illustrated the existence of four distinct phenotypes associated with the antimicrobial susceptibility profiles of the parental and their spontaneous mutant strains (Table 3). The first phenotype included no change of MICs for amoxicillin/clavulanic acid and ampicillin between the parental strains and their mutants (Table 3). The second phenotype showed a slight increase of MICs for ceftriaxone and gentamycin between the parental and their mutant strains (Table 3). The third phenotype was characterized by a variable change of MICs for tetracycline and streptomycin among the four *S.* Enteritidis parental strains and their spontaneous mutants (Table 3). For instance, the parental A-5 strain and its resistant and highly resistant mutant strains had the same MICs values of < 4 μg/mL for tetracycline. In contrast to this group of A-5 strains, parental A-7 strain exhibited an MIC of < 4 μg/mL; however, its resistant and highly resistant mutants acquired MICs of 8 μg/mL and 32 μg/mL, respectively, for the same antibiotic. The fourth phenotype was characterized by an increase in the MICs between the parental strains and their resistant and highly resistant mutants (Table 3). The MICs values for amikacin, chloramphenicol, cefoxitin, azithromycin, and ceftiofur of all of the *S.* Enteritidis strains have been accurately correlated to their ciprofloxacin MICs. The highest MICs values for these five antibiotics demonstrated ciprofloxacin highly resistant strains, whereas the lowest MICs values for the same five antibiotics exhibited the ciprofloxacin susceptible (i.e., parental) strains.

**Table 3:** Antimicrobial susceptibilities profiles of four parental *S*. Enteritidis strains and their resistant and highly resistant ciprofloxacin mutants.

FQ Susceptibility groups												
Strain IDs	Susceptible				Resistant				Highly resistant			
	A-5	A-7	A-21	A-33	A-5	A-7	A-21	A-33	A-5	A-7	A-21	A-33
**No change of MIC (μg/mL)**												
Amoxicillin/ Clavulanic acid	<1/0.5	<1/0.5	<1/0.5	<1/0.5	<1/0.5	<1/0.5	<1/0.5	<1/0.5	<1/0.5	<1/0.5	<1/0.5	<1/0.5
Ampicillin	<1	<1	<1	<1	<1	<1	<1	<1	<1	<1	<1	<1
**Slight change of MIC**												
Ceftriaxone	<0.25	<0.25	<0.25	<0.25	0.5	1	0.5	1	0.5	1	1	1
Gentamicin	2	2	1	4	4	2	1	2	4	1	4	2
**Variable change of MIC**												
Tetracycline	<4	<4	<4	<4	<4	8	<4	8	<4	32	8	<4
Streptomycin	64	64	64	>64	>64	>64	64	>64	>64	>64	64	>64
**Increase of MIC (Cross-resistance**)												
Amikacin	8	32	8	16	32	>64	>64	>64	>64	>64	>64	>64
Azithromycin	0.12	0.25	0.12	0.25	4	8	8	8	8	>16	>16	>16
Chloramphenicol	4	8	4	4	8	32	16	32	32	>32	>32	32
Cefoxitin	2	4	1	2	8	>32	>32	>32	32	>32	>32	>32
Ceftiofur	1	1	1	1	2	8	4	8	4	8	8	8

### Mutations Associated With the Ciprofloxacin Resistant and Highly Resistant Strains

**FIGURE 1:**
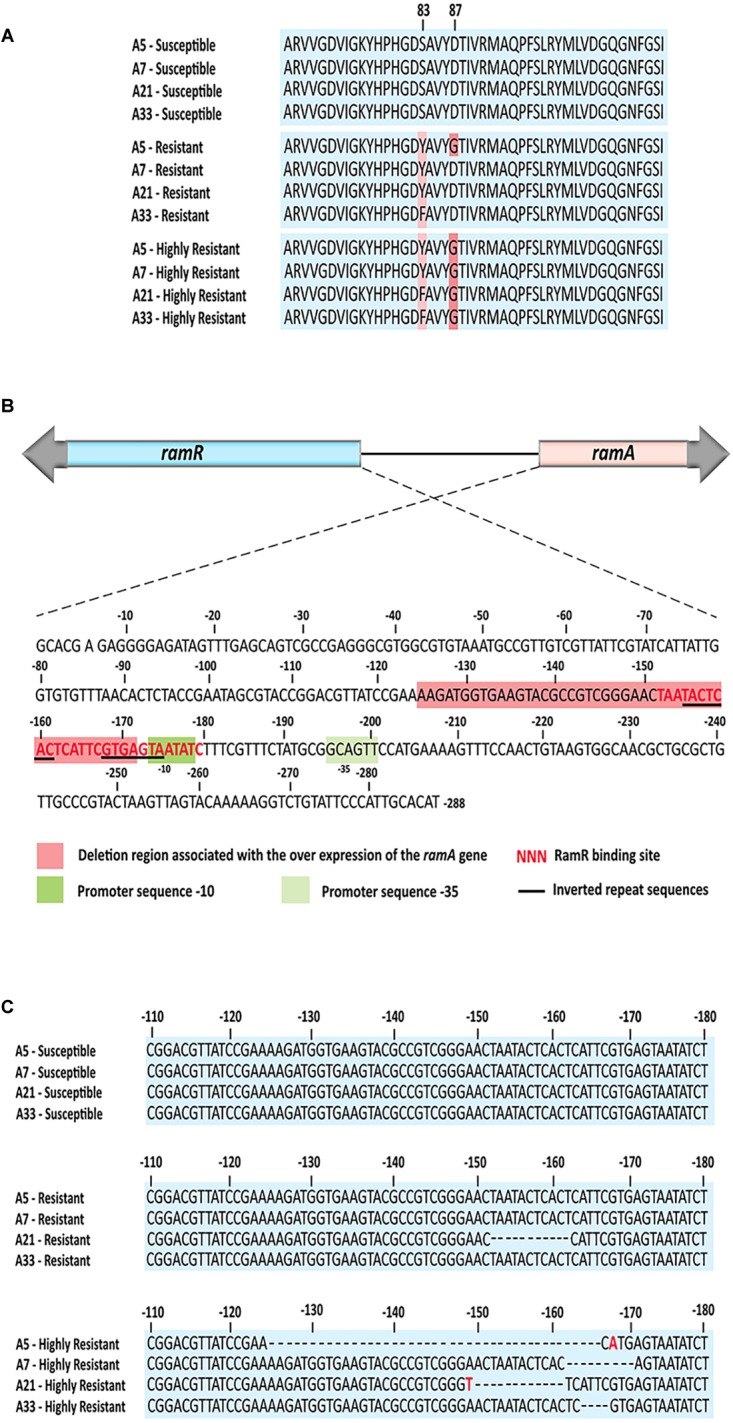
The common mutations associated with the ciprofloxacin resistant and highly resistant mutants. **(A)** Comparative amino acid analysis of the GyrA identified amino acid substitutions S83Y and S83F among all four resistant mutants as well as double amino acid substitutions S83Y, S83F and D87G among all four highly resistant strains and one resistant strain. **(B)** Genetic organization of the *ram* locus, showing orientations of the ramR and ramA gens, their intergenic region with the RamR binding site, the *ramA* promoter sequences, inverted repeat sequences and deletion region associated with the highly resistant mutants. **(C)** Nucleotide sequence alignments of the *ramRA* intergenic regions of all three ciprofloxacin susceptible groups. Dash lines indicate deletion mutations, while the red color of nucleotide indicates nucleotide base substitution. The RamR binding site for *S*. enterica was determined by Baucheron et al. (2011).

To identify mutations that contribute to the development of FQs-resistance (MIC from 8 to 16 μg/mL) and high resistance (MIC 2048 μg/mL), we sequenced the genes that encode topoisomerase II (*gyrA*, *gyrB*, *parC*, *parE*), efflux pump (*acrA*, *acrB*, *tolC*), efflux pump regulators (*ramR*, *ramA*), porins (*ompF*, *ompC*), extracytoplasmic stress response regulators (*rpoE*, *cpxR*), and liposaccharide component (*lpxA*) of the four parental strains along with their resistant and highly resistant mutants. No mutation was detected in the coding sequences of *gyrB*, *parE*, *acrA*, *acrB*, *tolC*, *ramA*, *ramR*, *ompF*, *ompC*, *rpoE*, *cpxR*, and *lpxA*. In addition, the entire promoter regions of the *acrA*, *acrB, tolC*, *rpoE*, and *cpxR* genes were intact, with no introduced mutations in either ciprofloxacin-resistant or ciprofloxacin-highly resistant mutants. A single non-synonymous substitution, G78D, was present in the *parC* of the highly resistant A-7 mutant strain. Another two amino acid substitutions, S80R and S80I, were present in the same gene of the resistant A-5 and highly resistant A-5 strains, respectively. In addition to these three amino acid substitutions, no other mutation was detected in the *parC* of the resistant A-7, resistant A-21, highly resistant A-21, resistant A-33, and highly resistant A-33 mutant strains. In contrast to the sporadic occurrence of mutations in *parC*, the *gyrA* was found to be a common target for the development of FQs resistance in the tested *S.* Enteritidis strains (Figure 1A and Table 2). The two FQs-resistant mutants, A-7 and A-21, acquired the same amino acid substitution, S83Y, the resistant A-33 mutant received another amino acid substitution, S83F, while the resistant A-5 mutant obtained two amino acid substitutions, S83Y and D87G (Table 2). The highly resistant mutants acquired a high-frequency mutation, the second amino acid substitutions, D87G (Table 2). The second high-frequency mutation associated with an extremely high level of ciprofloxacin resistance was identified in an intergenic region of *ramRA* (Figure 1B). This mutation was first identified among the resistant mutants, a single A-21 strain showed an 11-nt deletion (from 150 to 161 bp upstream of the *ramA*), which corresponds to the RamR binding site (Figure 1C and Table 2). This mutation progressed as the drug concentration increased and reached its high frequency among the highly resistant mutant strains. All the highly resistant mutant strains acquired a deletion that disrupted the RamR binding site (Figure 1C). To further investigate the effect of the *ramRA* intergenic deletion, the *ramRA* haplotype of the highly resistant A-5 strain was cloned into pTrc99-A and transformed into the parental A-5 strain. The complemented strain exhibited resistance to ceftiofur, amoxicillin/clavulanic acid and decreased susceptibility to azithromycin, tetracycline and ciprofloxacin, compared to the same strain transformed with the empty vector (Table 4). It is interesting that the A-5 parental strain complemented with the pTrc99A::ramRA vector could not develop a full ciprofloxacin resistance but only a decreased susceptibility to this antibiotic.

### Gene Expression Analysis of the *acrAB-tolC* Efflux Pump, Its Regulators *ramRA*, Outer Membrane Components and Extracytoplasmic Regulators

**FIGURE 2:**
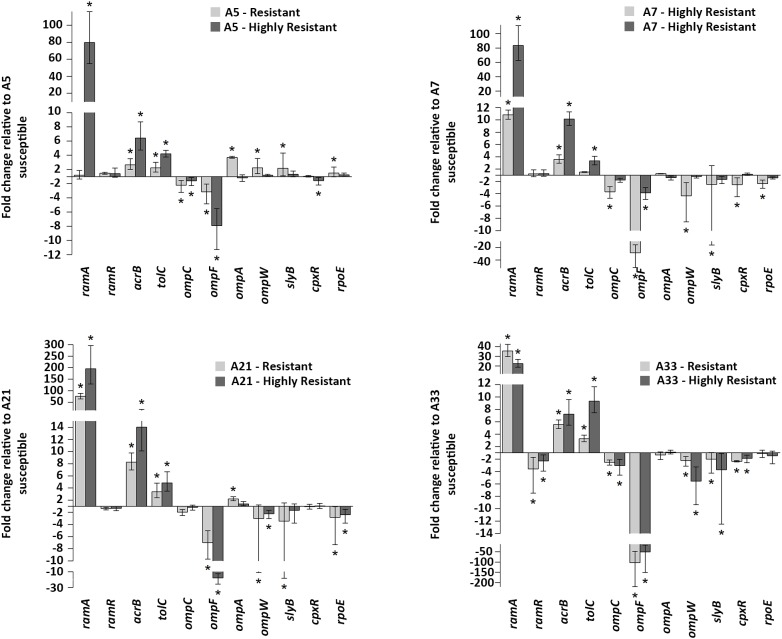
Gene expressions. mRNA expression levels of *acrB*, *tolC* (efflux pump), *ramR*, *ramA* (regulators of the *acrAB*-*tolC* efflux pump), *ompF*, *ompC*, *ompA*, *ompW*, *slyB* (porins) and *rpoE*, *cpxR* (extracytoplasmic stress response regulators) in all ciprofloxacin susceptible groups. Values on the *y* axis are relative expression levels (fold change) normalized against levels in the resistant and highly resistant strains. The data correspond to the mean values of three biological replications. *Error bars* correspond to the standard deviation. *Asterisks* indicate statistically significant differences (^∗^*p* < 0.05) in *t*-tests.

To compare the effect of the ciprofloxacin treatment on the gene expressions of the parental, resistant, and highly resistant strains, a subinhibitory concentration of this antibiotic for the A-5 parental strain was used. The selected ciprofloxacin concentration did not have any effect on the growth rate of this strain (data not shown). The expressions of the efflux pump genes, *acrA*, *acrB*, *tolC*, efflux pump regulators, *ramR*, *ramA*, extracytoplasmic regulators, *rpoE*, *cpxR*, and genes encoding porins, *ompA*, *ompW*, *ompF*, *ompC*, *slyB* for the parental strains and their resistant and highly resistant mutants are shown in Figure 2. Most notably, the expression of the *ramA* gene significantly increased in the all-highly resistant and resistant mutants except for the A-5 resistant mutant (Figure 2). The upregulation of the *ramA* gene ranged from 205 folds in the highly resistant A-21 strain to 10 folds in the resistant A-7 strain. Both genes associated with the *acrAB-tolC* efflux pump, *acrB* and *tolC*, showed significant (*p* < 0.05) levels of expression across the highly resistant and resistant strains. In general, the *acrB* exhibited a higher level of upregulation compared to that of the *tolC* gene. The highly resistant strains exhibited a higher expression of genes, *acrB* and *tolC*, compared to their resistant counterparts (Figure 2). In contrast to the efflux pump, gene encoding outer membrane porin F, *ompF*, showed significant (*p* < 0.05) downregulation across the all-highly resistant and resistant mutant strains (Figure 2). Another gene-encoding outer membrane protein C, *ompC*, was downregulated in all the resistant and highly resistant mutants but at a less significant rate compared to that of the *ompF* (Figure 2). The other five genes, *ompA*, *ompW*, *slyB*, *cpxR*, and *rpoE*, displayed a strain dependent pattern of expression (Figure 2).

**Table 4:** Differences in antimicrobial susceptibility of 5A susceptible strain and its counterparts complemented with an empty pTrc99A and pTrc99A::*ramRA* vectors.

	MIC (μg/mL)		
Drug	A-5 Susceptible	A-5 Susceptible /pTrc99A	A-5 Susceptible /pTrc99A::*ramRA*
Azithromycin	0.12	0.25	8
Amoxicillin/clavulanic acid	<1/0.5	8/4	>32/16
Ceftiofur	1	1	>8
Tetracycline	<4	<4	32
Ciprofloxacin	0.015	0.08	1

### Bacterial Growth Assay

**FIGURE 3:**
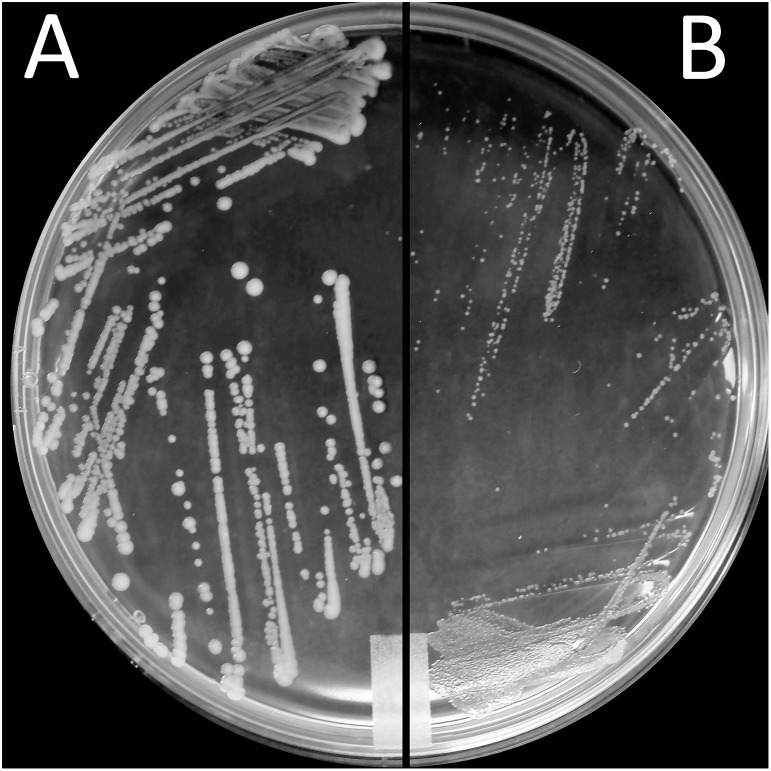
Small colony variant phenotype. Morphological appearance of *S*. Enteritidis colonies on LB agar after incubation for 24 h at 37°C. Shown are colonies of **(A)** parental (FQs susceptible) A-21 strain and **(B)** small colony variants (SCV) of A-21 spontaneous (FQs highly resistant) mutant strain.

**FIGURE 4:**
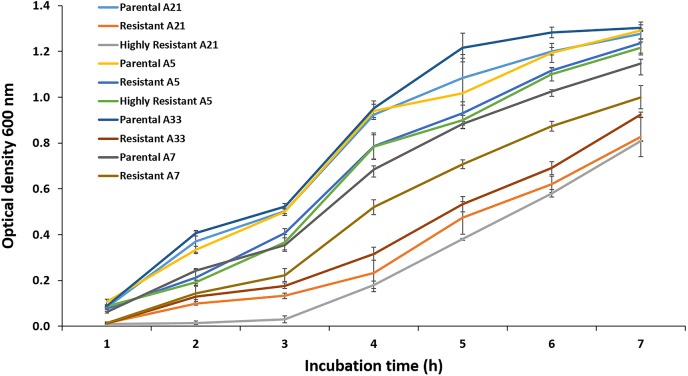
Growth assay. The growth kinetics for the A-5, A-7, A-21, and A-33 parental and their resistant mutants were measured during the exponential growth phase. Also the growth curves were measured for the A-5 and A-21 highly resistant strains. The data correspond to the mean values of three biological replications.

Exposure to a high concentration of ciprofloxacin (e.g., 40 μg/mL) resulted in the acquisition of the SCV phenotype among the highly resistant A-7, A-21, and A-33 mutants (Figure 3A,B). To characterize the growth kinetics of the SCV and non-SCV mutants, we compared the growth curves of the A-21 and A-5 parental strains with their highly resistant mutants. Our data showed that the growth curve of the highly resistant A-21 mutant strain was significantly impaired (*p* < 0.05) compared to the growth curve of its parental strain (Figure 4), whereas the A-5 highly resistant mutant did not show significant growth alteration compared to its parental strain (Figure 4). To determine the effect of multiple QRDR mutations on *S*. Enteritidis fitness, we compared the growth curves of the A-5 resistant strain (i.e., GyrA S83Y, D87G; ParC S80R) and its A-5 parental strain (i.e., no QRDR mutations). There was no significant difference in the growth rate between these two strains (Figure 4). Next we compared the growth curves of the A-7 (GyrA S83Y), A-21 (GyrA S83Y) and A-33 (GyrA S83F) resistant strains with their parental strain (i.e., no QRDR mutations). The A-7, A-21 and A-33 resistant strains showed an impaired growth compared to those of other three strains (Figure 4).

## Discussion

Resistance to FQs class of antibiotics can be acquired vertically through *de novo* mutations and horizontally via the introduction of FQs resistance genes. The vertical evolution of FQs resistance can be very fast to allow the bacterial organism to develop resistance during a single antimicrobial treatment, which can lead to the treatment failure and death of a patient. Recently, Blair et al. (2015), examining the genomes of pre- and post-therapy isolates of *S*. Typhimurium from a patient who failed antimicrobial therapy, revealed a single mutation in the efflux pump gene, *acrB*, which was acquired during the antimicrobial treatment. This novel G288D substitution in the drug-binding pocket of AcrAB-TolC multidrug efflux pump caused resistance to the FQs group of antibiotics but susceptibility to the other drugs (e.g., doxorubicin and minocycline), underscoring the complexity and importance of vertical evolution in the development of antimicrobial resistance. Understanding the dynamics of drug-resistant mutations and their interactions and stress adaptive physiology of bacterial organism is important not only for rational drug design but also for predicting the evolution of antibiotic resistance in clinical settings. In the current study, using four ciprofloxacin susceptible *S*. Enteritidis clinical isolates in combination with stepwise selection, we determined the type and dynamics of the mutations associated with resistance and high-level resistance to ciprofloxacin as well as the stress adaptive response of the *S*. Enteritidis FQ mutant strains.

Among 14 genes, targeting topoisomerase II (*gyrA*, *gyrB*, *parC*, *parE*), the AcrAB-TolC multidrug efflux pump (*acrA*, *acrB*, *tolC*), the efflux pump regulators (*ramR*, *ramA*), porins (*ompF*, *ompC*), extracytoplasmic stress response regulators (*rpoE*, *cpxR*), and lipopolysaccharide component (*lpxA*), the first high-frequency mutation in all the four resistant *S*. Enteritidis strains occurred in the QRDR of *gyrA*, resulting in S83Y and S83F substitutions. Among the population of the resistant strains, another amino acid substitution, S80R, in the *parC* was found only in the A-5 strain, indicating the sporadic (i.e., mutation that occurred in one or two strains) nature of this mutation. As the resistance progressed, the second high-frequency amino acid substitution, D87G, was identified in the *gyrA* among the all-highly resistant strains. Among the group of the highly resistant strains, another sporadic amino acid substitution, G78D, occurred in the *parC* of the A-7 strain, confirming that the *parC* is not the primary target of ciprofloxacin resistance in *S*. Enteritidis.

It has been shown that, in *S.* Typhimurium, the regulator RamA plays a key role in the activation of AcrAB-TolC multidrug efflux pump (Abouzeed et al., 2008; Nikaido et al., 2008). O’Regan et al. (2009) showed that inactivation of AcrAB-TolC leads to an increased susceptibility to ciprofloxacin and other non-quinolone antibiotics, clearly indicating that the AcrAB-TolC efflux pump plays a role in fluroquinolone resistance and MDR in *S*. Enteritidis. Therefore, we examined the mutations in the coding and non-coding regions of the *ramRA* regulator in all-*S.* Enteritidis resistant and highly resistant mutant strains. The first mutation associated with the *acrAB-tolC* efflux pump was identified as an 11 bp deletion in the *ramRA* intergenic region, affecting the RamR binding site. As the exposure of *S*. Enteritidis to drug concentration increased, deletions in the *ramRA* intergenic regions, affecting the RamR binding site, intensified, resulting in a maximum frequency among the group of highly resistant mutants. To determine the effect of this mutation on antimicrobial susceptibility of *S*. Enteritidis serovar, we complemented the A-5 parental strain with the recombinant plasmid, which contains the *ramRA* haplotype of its highly resistant A-5 mutant strain. The complemented parental strain showed complete resistance to ceftiofur and amoxicillin/clavulanic acid as well as decreased susceptibility to azithromycin and tetracycline, compared to its counterpart complemented with an empty plasmid. This experimental finding clearly points out that the deletion in the *ramRA* intergenic region, affecting the RamR binding site, plays a role in the development of multidrug resistance in *S*. Enteritidis. It is interesting that the complemented parental strain developed resistance against amoxicillin/clavulanic acid, whereas the highly resistant mutant exhibited susceptibility to the same drug. This discrepancy could be explained by the difference in the fitness landscape of these two organisms. Hartl (2014) showed that there are synergistic and antagonistic interactions among the resistant-conferring mutations, and that the comprehensive measurements of resistance should be based on the sum of these interactions. It is possible that the *ramRA* haplotype in the highly resistant strain faced antagonistic mutations, whereas these mutations were absent in the complemented parental strain.

To gain an insight into the adaptive response of *S*. Enteritidis to high concentrations of ciprofloxacin, we determined the expression patterns of a wide range of genes in the susceptible, resistant, and highly resistant groups of *S*. Enteritidis. Most notably, the expression of genes, encoding the *acrAB*-*tolC* efflux pump and its activator *ramA*, were significantly upregulated in almost all the strains of the resistant and highly resistant groups. In contrast, the gene, encoding porin *ompF*, was significantly downregulated in each strain of the resistant and highly resistant groups. Several studies that investigated *ompF* expression in FQs-resistant *Salmonella* illustrated the unclear role of this porin in contribution to FQs resistance (Giraud et al., 2000; O’Regan et al., 2009). Our data shown that the downregulation of *ompF* is a common adaptive response of *S*. Enteritidis, while the downregulation of *ompC* was less profound and more strain-dependent, suggesting its minor role in the development of *Salmonella* FQs resistance. It has been shown that mutations in the *marR* regulator result in both an increase in *acrB* expression and a decrease in *ompF* expression (Randall and Woodward, 2002; Hooper and Jacoby, 2015). Our recent study showed that the *rpoE* sigma factor also inhibits the synthesis of outer membrane porins during the antimicrobial treatment of *Escherichia coli* O157 with a polycationic agent (Vidovic et al., 2018), further illustrating that the porin-mediated permeability plays a role in the development of resistance to various antimicrobial agents.

Comparing the expression of the genes and amino acid substitutions, the A-5 resistant strain showed a distinct characteristic compared to the rest of resistant mutants. Only the A-5 strain, once exposed to the subinhibitory concentration of ciprofloxacin did not up regulate the expression of the *ramA* gene. It is noteworthy to mention that the A-5 resistant strain acquired three amino acid substitutions (S83Y, D87G in GyrA and S80R in ParC), whereas other resistant mutants acquired one amino acid substitution at 83 position of GyrA. It has been documented that the number of QRDR amino acid substitutions has a detrimental effect on the stress adaptive physiology of several bacterial species, including *Staphylococcus aureus* (Horváth et al., 2012), *Klebsiella pneumoniae* (Tóth et al., 2014), and *Escherichia coli* (Johnson et al., 2015). Bacterial strains that acquire multiple QRDR mutations develop high-level resistance against FQs and preserve fitness (Fuzi et al., 2017). In contrast, strains with fewer QRDR mutations must relay on the FQs non-specific mechanisms, primarily on efflux, an energetically demanding mechanism, which has a direct influence on bacterial fitness (Fuzi, 2016). To determine the relationship between the energetically favorable QRDR mutations and bacterial fitness, we performed the growth assays using the A-5 resistant (multiple QRDR mutations) and its parental A-5 susceptible strain as well as the A-21 and A-33 resistant (single QRDR mutation) and their parental strains. Our data showed that the A-5 resistant strain has a similar growth curve as its parental strain, whereas the A-21 and A-33 resistant strains showed altered growth compared to its parental strain. Overall, this study provides evidence that *S*. Enteritidis with multiple QRDR mutations do not activate efflux to reach resistance to ciprofloxacin (4 μg/mL), while a strain with fewer QRDR mutations must activate efflux to reach the same MIC. Further, this stress adaptive response has an important effect on the overall fitness of *S*. Enteritidis.

Finally, this study revealed the existence of a small colony of variants (SCVs) phenotype among the resistant *S.* Enteritidis strains. These highly resistant strains of *S.* Enteritidis are characterized by a significant growth deficiency and small colony size compared to their parental strains. It has been demonstrated that the growth rate of an organism directly correlates to the antimicrobial resistance (Fuentes-Hernandez et al., 2015; Knopp and Andersson, 2018), with the more slowly replicating organisms being more resistant compared to their faster replicating counterparts. O’Regan et al. (2010) showed that in the absence of selective pressure, a spontaneous ciprofloxacin resistant *S*. Enteritidis strain after 20 passages reverted to its parental phenotype (e.g., reversal of all fitness cost except motility). They also showed that with increased fitness of the reverted strain ciprofloxacin susceptibility of this strain significantly increased. This physiological adaptation (i.e., growth rate) most likely represents another mechanism employed by *S.* Enteritidis to confer an extremely high level of resistance to FQs. It is interesting that only the A-5 mutants did not acquire SCV phenotype and showed altered growth compared to their parental strain. These strains preserved their original fitness while developing the high level of FQ resistance further indicating most likely the existence of unique compensatory mutations that maintain the high level of FQ resistance while maintaining the original strain fitness.

In summary, the development of FQs resistance among a population of clinical isolates of *Salmonella* spp. is of a great health concern. This study provides two important novel findings. The first, the deletions in the *ramRA* region, affecting the RamR binding site, and the amino acid substitution at position 87 of the GyrA are mutations significantly associated with the highly resistant *S.* Enteritidis strains, clearly illustrating their importance in conferring a high level of FQs resistance. The second, the deletion in the intergenic region of the *ramRA* operon provides resistance and reduced susceptibility to several antibiotics, including, ceftiofur, amoxicillin/clavulanic acid, azithromycin and tetracycline. Collectively, our study suggests complex interactions between mutations, bacterial adaptations, and FQs resistance.

## Author Contributions

SV conceived the study, carried out the experiments, and drafted the manuscript. RA carried out the experiments. AR performed the statistical analysis related to the gene expressions.

## Conflict of Interest Statement

The authors declare that the research was conducted in the absence of any commercial or financial relationships that could be construed as a potential conflict of interest.
