# Supplementary material for: Molecular and Physiological Characterization of Fluoroquinolone-Highly Resistant Salmonella Enteritidis Strains
Source: Front Microbiol. 2019 Apr 9;10:729. doi: 10.3389/fmicb.2019.00729 (PMC6465633; doi:10.3389/fmicb.2019.00729)
Supplement: Supplementary file 1 [file Table_1.DOCX]

**Table S1.** Primers used for the gene expression assay.

| **Gene** | **Name of primer** | **Forward primer sequence**  **(5’ – 3’)** | **Name of primer** | **Reverse primer sequence**  **(5’ – 3’)** |
| --- | --- | --- | --- | --- |
| *acrB* | *acrB* F | GGCAACCGTACTGGCTATTT | *acrB* R | GCGATGTTCTGTCGAATGACTA |
| *tolC* | *tolC* F | CCTCCATCAGCAGCATCAA | *tolC* R | TACGTGTACCGACCGAGTAA |
| *ramR* | *ramR* F | TGATGGTGTTTATGTCGGATGA | *ramR* R | TTCGAATCCCAGCGCAATA |
| *ramA* | *ramA* F | GTGCGTGAACGGAAGCTAAA | *ramA* R | GGTTGAACGTGCGGGTAAA |
| *ompC* | *ompC* F | GCGCAGTATTCTCAGACCTATAA | *ompC* R | ACCAAAGTCGAACTGGTACTG |
| *ompF* | *ompF* F | TGCAGAGTAAAGGTAAGCAGTT | *ompF* R | AGCAGGTTGAAACGGTAGTC |
| *ompA* | *ompA* F | CGTATCGGTTCTGACGCTTAC | *ompA* R | GACGGAATACCTTTGGAGATCAG |
| *ompW* | *ompW* F | CCTCTGTCTGGTACATGGATATTG | *ompW* R | GATAGCCTGCCGAGAACATAAA |
| *slyB* | *slyB* F | CCTCTGTCTGGTACATGGATATTG | *slyB* R | GATAGCCTGCCGAGAACATAAA |
| *rpoE* | *rpoE* F | GGCCTGAGCTATGAAGAGATAG | *rpoE* R | CCTGATAAGCGGTTGAACTTTATT |
| *cpxR* | *cpxR* F | CGGAATTCACCCTGCTCTATTT | *cpxR* R | CCAGCACTTCCTGGCTTAAA |
| *gapA* | *gapA* F | ACTTCCGTGTTCGATGCTAAA | *gapA* R | GGTCCAGTACTTTGTTGGAGTAA |
